# Only a Single Taxonomically Restricted Gene Family in the *Drosophila melanogaster* Subgroup Can Be Identified with High Confidence

**DOI:** 10.1093/gbe/evaa127

**Published:** 2020-06-26

**Authors:** Karina Zile, Christophe Dessimoz, Yannick Wurm, Joanna Masel

**Affiliations:** 1 Division of Biosciences, University College London, United Kingdom; 2 Swiss Institute of Bioinformatics, Lausanne, Switzerland; 3 Department of Computational Biology, University of Lausanne, Switzerland; 4 Center for Integrative Genomics, University of Lausanne, Switzerland; 5 Department of Genetics, Evolution and Environment, University College London, United Kingdom; 6 Department of Computer Science, University College London, United Kingdom; 7 School of Biological and Chemical Sciences, Queen Mary University of London, United Kingdom; 8 Alan Turing Institute, London, United Kingdom; 9 Department of Ecology and Evolutionary Biology, University of Arizona

**Keywords:** taxonomically restricted genes, de novo gene birth, Drosophila, genome evolution, new genes, de novo genes

## Abstract

Taxonomically restricted genes (TRGs) are genes that are present only in one clade. Protein-coding TRGs may evolve de novo from previously noncoding sequences: functional ncRNA, introns, or alternative reading frames of older protein-coding genes, or intergenic sequences. A major challenge in studying de novo genes is the need to avoid both false-positives (nonfunctional open reading frames and/or functional genes that did not arise de novo) and false-negatives. Here, we search conservatively for high-confidence TRGs as the most promising candidates for experimental studies, ensuring functionality through conservation across at least two species, and ensuring de novo status through examination of homologous noncoding sequences. Our pipeline also avoids ascertainment biases associated with preconceptions of how de novo genes are born. We identify one TRG family that evolved de novo in the *Drosophila melanogaster* subgroup. This TRG family contains single-copy genes in *Drosophila simulans* and *Drosophila sechellia*. It originated in an intron of a well-established gene, sharing that intron with another well-established gene upstream. These TRGs contain an intron that predates their open reading frame. These genes have not been previously reported as de novo originated, and to our knowledge, they are the best *Drosophila* candidates identified so far for experimental studies aimed at elucidating the properties of de novo genes.

## Introduction

Some genes are present only in one clade, and are therefore called taxonomically restricted genes (TRGs). They are also referred to as *orphans* or simply novel genes. Some of these may have originated de novo. We use the original version of the selected effect definition of function (Linquist et al. 2020) to determine when a sequence becomes a protein-coding gene. This means that de novo birth occurs at the moment beyond which a mutation leading to loss of the protein product would have a negative effect on fitness. For the birth to occur, a de novo TRG needs not only the amino acid sequence itself but also the right environment and expression regulation pattern, to confer an advantage to the organism. Protein-coding genes may evolve de novo from noncoding regions (McLysaght and Guerzoni 2015;Vakirlis et al. 2018), in alternative frames of established genes (Guan et al. 2018; Willis and Masel 2018), or as a result of genome rearrangement (Chen et al. 2015; Stewart and Rogers 2019).

The research enterprise is biased toward studying ancient gene families with homologs across multiple model organisms, and so the properties and evolutionary dynamics of young TRGs are not well understood. TRGs are likely to include proteins with as yet undocumented functions and, especially in the case of de novo genes, new protein domains or other structural forms that are yet to be discovered (Bungard et al. 2017). Mounting evidence suggests that TRGs can acquire important functions. For example, a TRG in the tardigrade *Ramazzottius varieornatus* produces a protein that protects DNA and improves radio-tolerance (Hashimoto et al. 2016). TRGs in *Hymenoptera* are implicated in the speciation of parasitoid wasps and in the production of diverse venoms characteristic of this clade (Werren et al. 2010). Albertin et al. (2015) identified numerous cephalopod-specific genes and were able to find hints about their diverse functions based on their tissue-specific expression profiles. These examples remain anecdotal since functional characteristics of TRGs cannot be inferred computationally due to the lack of homologs outside a specific clade.

Most previous studies aimed at elucidating properties and rates of emergence of de novo TRGs have used an approach known as “phylostratigraphy” that focuses on protein-coding genes with protein homologs within a particular clade and no detectable homology outside that specific clade. This approach is incapable of discriminating between de novo genes and highly diverged copies of well-established genes (Weisman et al. 2020). Hence, the properties of “young genes” reported in these studies are averages computed across the two groups, and risk attributing to TRGs properties that instead reflect the disappearance of the ability to detect homology. For example, most of the studies reported that new genes tend to be shorter (Wissler et al. 2013; Zhao et al. 2014; Ruiz-Orera et al. 2015; Sun et al. 2015) and evolve faster than well-established genes (Domazet-Loso 2003; Toll-Riera et al. 2008; Donoghue et al. 2011). It is a priori plausible that TRGs have these properties, but phylostratigraphy does not provide clear evidence to support this claim. It is harder to detect homology for shorter and/or faster evolving genes, and this is sufficient to explain at least the qualitative direction of the observed trend. Including synteny information in the phylostratigraphy analysis changes the inferred gene ages (Arendsee et al. 2019), demonstrating that by itself the phylostratigraphy approach is not sufficient.

Related to this are substantive disputes about the frequency of de novo gene birth (Casola 2018), even though the existence of extremely well-documented case studies (Cai et al. 2008; Baalsrud et al. 2018) has made indisputable the qualitative claim that de novo gene birth is ongoing. Vakirlis et al. (2020) used synteny conservation to show that genes originate de novo from ancestral noncoding sequences as well as via divergence from ancestral genes. More quantitatively, synteny-based methods suggest that sequence divergence is not the main source of orphan genes (Vakirlis et al. 2020).

There are also compelling arguments for the plausibility of de novo gene birth. Purifying selection is expected to screen occasionally translated open reading frames (ORFs) in a way that makes them more viable as raw material (Wilson and Masel 2011). The physicochemical properties and secondary structures of evolved and random sequences are very similar, and randomly created sequences can be tolerated in vivo by *Escherichia coli* (Tretyachenko et al. 2017). Indeed, Neme et al. (2017) showed that at least two noncoding and one protein-coding gene could be selected from around a million randomly generated sequences (mimicking de novo evolution) in lab conditions. Although the beneficial nature of these genes is disputed (Weisman and Eddy 2017; Knopp and Andersson 2018), Knopp et al. (2019) similarly selected three random peptides conferring antibiotic resistance. At minimum, substantial tolerance clearly exists.

Although these arguments apply to de novo gene birth overall, the only way to be confident that a particular putative TRG is not merely a rapidly evolving gene duplicate is to find evidence of how it emerged. If we can identify homologous DNA region(s) in the species outside the clade from which a gene has emerged (i.e., the outgroup species), if these DNA regions are noncoding, and if we can rule out pseudogenization in this outgroup via synteny-based evidence of absence in more distant outgroups, then we have the evidence that the gene is specific to this particular clade, as well as information about the nature of the origination process. When a putative TRG has simply diverged beyond detection of its protein-coding homologs, no homology in noncoding sequence will be detectable either (although a syntenic homologous-coding sequence [CDS] may be found upon close scrutiny), and so a false-positive de novo gene identification will be avoided.

A false-positive could, however, arise from a horizontal gene transfer followed by pseudogenization in one lineage. Fortunately, such cases can often be excluded when homology to the donor clade is detectable. Both lack of donor sequence and pseudogenization in a member of the focal clade are required to generate such a false-positive, a scenario that in combination should be reasonably rare.

One important scenario to consider is when, following a gene duplication, the ortholog in the outgroup is lost or diverges beyond detectable homology. It is therefore important to consider all likely homologous DNA regions in outgroup species, not only the single-most likely region. One way to do this is to check whether the identified region in the outgroup species is homologous to any other regions in that genome. This is made relatively easy when the duplicated DNA region contains flanking, better-conserved genes, such that local synteny information can be exploited.

Even with synteny, detecting homologous noncoding sequences can be difficult. Noncoding regions of the genome are either under little evolutionary constraint, or under constraint very different from that of protein-coding regions, depending on their function or lack thereof. What constraint they have might apply to very general properties rather than to specific nucleotides at specific positions, and hence might not be enough to prevent rapid degradation of sequence similarity (Frigola et al. 2017). This means that it is necessary to confine analysis to closely related genomes in order to identify evolutionary origins of TRGs. A measure of “evolutionary traceability” of a protein family can quantify the evolutionary distance beyond which homologous proteins can no longer be identified (Jain et al. 2019). No similar metric exists for homologous noncoding DNA regions, but it is prudent to stick to closely related species.

Some analyses restrict their search for putative TRGs to the set of already-annotated protein-coding genes. Gene annotations are based largely on ORF length, transcription, and homology to known genes. Hence, a short TRG that has no previously known homologs is likely to be missed by an annotation algorithm, despite the fact that TRGs are expected a priori to be short. An alternative approach is to start with all ORFs present in the genome and exclude the ones that have no evidence for being functional. Previous studies used different types of evidence of functionality: Blevins et al. (2017) analyzed deep RNA sequencing and ribosome profiling data, Ruiz-Orera et al. (2018) combined that with proteomics data and single-nucleotide polymorphism analysis, whereas Vakirlis et al. (2018) developed a logistic regression classifier trained on coding and noncoding sequences using such properties as codon frequency, hydrophobicity and aromaticity scores, and structural predictions (secondary structures, transmembrane, and disordered regions). However, TRGs are expected to have a narrow expression profile (Wu and Knudson 2018) and they may have sequence properties distinct from well-studied protein families. There is thus a trade-off between false-positives (nonfunctional ORFs) and false-negatives (true TRGs excluded from the analysis). Beginning with annotated protein-coding genes tilts the balance toward false-negatives, whereas beginning with all ORFs tilts it toward false-positives. Regardless of how stringent or relaxed the requirements for evidence of functionality are, the resulting set of putative TRGs is unlikely to be both high confidence and exhaustive, limiting the potential for novel biological insights.

To advance our knowledge about de novo TRGs, resource-intensive experimental investigations of the most promising candidates are required, including knockout studies and structural biology experiments. Candidates need to be chosen from studies that prioritize avoiding false-positives over avoiding false-negatives. For example, BSC4, which is found only in *Saccharomyces cerevisiae*, has synthetic lethal knockouts (Cai et al. 2008). This strong functional evidence made it a good candidate for structural biology experiments, which showed that it folds to a partially specific 3D structure (Bungard et al. 2017). Absent such direct experimental data as synthetic lethal screens, the best indication of functionality is sequence conservation between several species (Graur et al. 2013), which is by definition unavailable for single-species TRGs, even when they are functional.

Several studies have focused on identifying the evolutionary origins of putative TRGs in primates, insects, and rosids, as a way of confirming their de novo nature (Toll-Riera et al. 2008; Zhou et al. 2008; Donoghue et al. 2011; Wissler et al. 2013; Sun et al. 2015). Unfortunately, these studies extensively ruled out TRG candidates based on thinly justified a priori assumptions about TRGs, in some cases discarding up to 61% of candidate genes (Vakirlis et al. 2018). For example, one study excluded genes with more than one coding exon because “it is difficult to distinguish the absence of coding potential due to frame-shifts and stop codons from the alternative explanation of evolutionary change of intron–exon boundaries” (Guerzoni and McLysaght 2016), perhaps also believing that the evolution of both a long ORF and an intron splicing signal is highly improbable (Knowles and McLysaght 2009). Interestingly, other studies excluded single-coding exon genes, either to avoid promoter- or enhancer-associated transcripts (PROMPTS and eRNAs) (Ruiz-Orera et al. 2015), or to avoid possible contamination of TEs incorrectly annotated as genes (Toll-Riera et al. 2008). Similarly, many studies excluded genes whose length is below a certain threshold (Yang and Huang 2011), genes with compositions too far from (Linquist et al. 2020) an average established protein-coding gene, and genes that are evolving too fast (Vakirlis et al. 2018). In perhaps the most extreme case, Casola (2018) excluded TRG candidates which are present in several copies in a genome due to a belief that young genes could not have had the time to duplicate.

Once they have identified TRGs, a second major limitation of studies focused on establishing the mechanism of origination is testing hypothesized mechanisms sequentially instead of looking holistically at the evidence available for each of the genes to establish their evolutionary origin. De novo protein-coding genes might be born within functional ncRNA, within introns or alternative frames of older protein-coding genes, or from intergenic sequences. Despite our desire to classify new genes into discrete categories, the evolutionary journey from an ancestral sequence to a new protein-coding gene might involve multiple steps, or vary along the gene’s length. For example, TRGs might contain both previously noncoding sequences and fragments of well-established genes. McLysaght and Hurst (2016) proposed the classification of TRGs into several groups based on the proportion of the sequence that has previously been under natural selection for protein-coding properties. However, the distinction can blur, for example, if previously protein-coding genes are pseudogenized or rearranged into noncoding sequence (see review by Balakirev and Ayala [2003]), and are then resurrected as part of a TRG. Although pre-existing transcription may obviously be an advantage, most of the genome is likely to be transcribed across relatively short evolutionary time in at least one cell type (Neme and Tautz 2016). Nonfunctional transcripts have been hypothesized to be a reservoir of genomic raw material that can increase organisms’ ability to adapt (Brosius 2005). On the other hand, their GC content makes ORFs from them more ordered and hence less suitable as raw material than for example the alternative reading frames of existing genes (Ángyán et al. 2012; Wilson et al. 2017; Casola 2018).

**Fig. 1.**
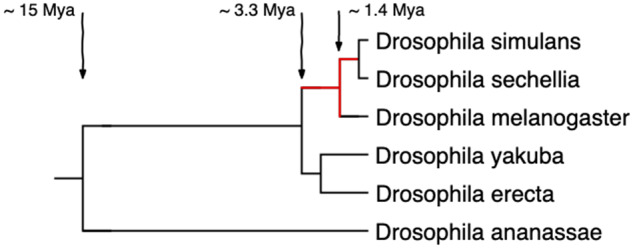
Species tree of the *Drosophila melanogaster* subgroup. Branch lengths correspond to divergence time estimates by Obbard et al. (2012). We looked for TRGFs that emerged during the evolutionary time marked in red, that is, between ∼0.5 and ∼3.3 Ma. We ultimately confirm one TRGF shared only by *D. simulans* and *D. sechellia*, that is, that originated between ∼0.5 and ∼1.4 Ma.

Here, we aim to identify high-confidence protein-coding genes that emerged de novo, hoping to provide a good starting point for experimental investigation. We focus on the *Drosophila melanogaster* subgroup, which is not only experimentally tractable but also has compact genomes of ∼140 Mb, and genome assemblies of five closely related species that range in quality from good to excellent. We look for taxonomically restricted gene families (TRGFs) that emerged after the split of the *simulans–sechellia–melanogaster* clade from the *yakuba–erecta* clade and before the speciation of *Drosophila simulans* and *Drosophila sechellia* (fig. 1). We use conservative but strongly justified criteria to identify putative de novo genes among annotated protein-coding genes that have homologs in at least two of the three species in the *simulans–sechellia–melanogaster* clade. By focusing on TRGFs instead of singleton TRGs, we hope to avoid genome sequencing and assembly artifacts. We used ORF conservation across two to three species as a proxy for functionality under the selected-effect definition (Graur et al. 2013), as the half-life of a nonfunctional ORF is small given the probability of acquiring a stop codon by chance. A d*N*/d*S* signal of selection would be still stronger evidence for functionality, but short sequences in three closely related species do not contain enough information to reliably distinguish deviations from d*N*/d*S* = 1. By identifying the evolutionary origins of TRGs that have passed our conserved-ORF criterion for functionality, which we do by examining the homologous DNA region in the most closely related species that lack(s) the ORF, we aim to both validate their de novo origin (providing vetted experimental candidates) and improve our understanding of how de novo genes emerge.

## Results

**Fig. 2.**
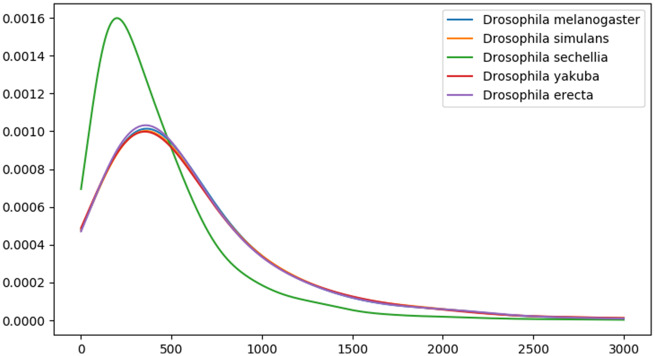
Protein length distributions in five *Drosophila melanogaster* subgroup species.

The five species we study in the *D. melanogaster* subgroup (*D. melanogaster*, *D. simulans*, *D. sechellia*, *Drosophila yakuba*, and *Drosophila erecta*) had a common ancestor ∼3.3 Ma (Obbard et al. 2012) (fig. 1). Each has a genome of ∼140 Mb containing ∼14,000 protein-coding genes. There is no evidence of major segmental genome duplications in this clade, reducing complications in identifying homologous noncoding sequences. The genome assembly for *D. sechellia* is highly fragmented, as confirmed by N50 metric and a BUSCO (Waterhouse et al. 2018) estimate that ∼8% of the genes likely present in the genome are missing from the assembly (table 1). The quality of the *D. sechellia* genome assembly leads to a different distribution of annotated protein lengths compared with other species in this clade (fig. 2). For this reason, we should be especially cautious of inferring anything based on absence from *D. sechellia*.

**Table 1:** Genome Assemblies of *Drosophila melanogaster* Subgroup Species Used in This Study

Species (RefSeq assembly accession)	Assembly Size (Mb)	Molecule Count	N50 (Mb)	Proteins	BUSCO (%)
*D. simulans* (GCF_000754195.2)	124.96	7	0.45	14179	98.6
*D. sechellia* (GCF_000005215.3)	166.59	1	0.042	16467	92.2
*D. melanogaster* (GCF_000001215.4)	116.52	8	19.48	13916	99.3
*D. yakuba* (GCF_000005975.2)	165.71	8	0.12	14824	98.4
*D. erecta* (GCF_000005135.1)	152.71	0	0.45	13605	99

Based on the OMA homology inference algorithm (Altenhoff et al. 2018), these five *D. melanogaster* subgroup species contain 14,149 gene families. Among the inferred gene families there were 205 families with genes in at least two of the species in the *simulans–sechellia–melanogaster* clade and no genes from species outside the clade. Protein sequence similarity searches against the RefSeq database revealed diverged homologs outside the clade for 170 of these families. We used sequence similarity searches in nucleotide space to identify homologous DNA regions corresponding to the 35 putative TRGFs in all five genomes. Out of these 35 families, 18 contained conserved but unannotated ORF(s) covering ≥50% of the putative TRGF ORF in at least one of the *yakuba–erecta* clade species, indicative of an earlier origin of these TRGs. A conserved ORF in an outgroup was considered strong evidence that the gene family originated before the speciation of the clade. We were unable to obtain a continuous alignment of inferred homologous DNA regions in the *yakuba–erecta* clade for five putative TRGFs. It is unknown whether this is due to genome rearrangements and the lack of sequence conservation or simply because the true homologous DNA regions are missing from the genome assemblies. Only the 12 putative TRGFs for which we were able to obtain a continuous alignment of homologous DNA regions in all five species and show that the ORFs were only present in the *simulans–sechellia–melanogaster* clade were considered in further analyses.

Manual examination of genome annotations revealed problems and inconsistencies with ten putative TRGF annotations, such that we were uncertain about the nature or location of the ORF. For example, some of the gene families were missing a start codon, had annotated exons that overlapped in alternative frames, exons misaligned with splicing signals, or inconsistent start/stop codons and/or splicing signals across species. These putative TRGF were removed from further analysis as they did not satisfy our requirement for a conserved ORF in more than one independently annotated species.

**Fig. 3.**
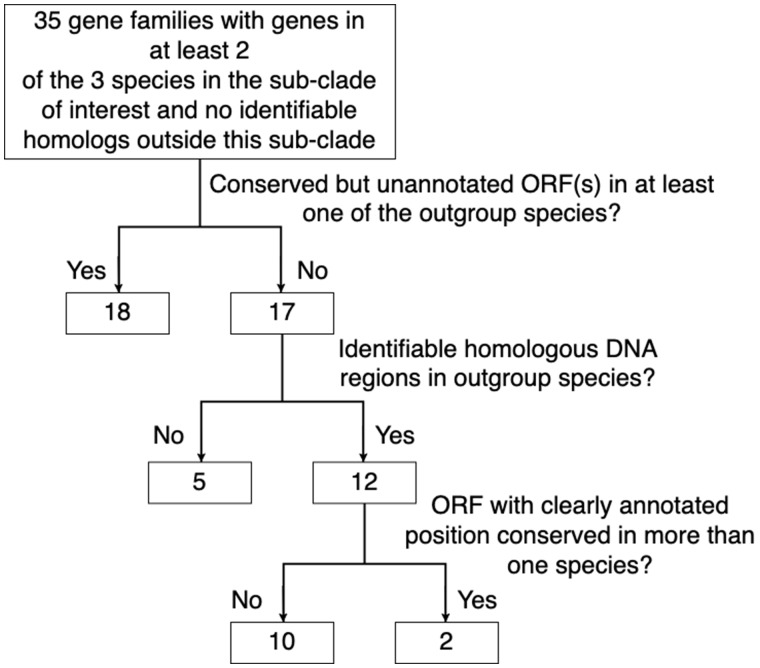
The elimination of TRGFs with either evidence of being false-positive, or with insufficient evidence available.

To infer the evolutionary origins of the two putative TRGFs that remained following these filters to remove potential false-positives (summarized in fig. 3), we looked at the homologous noncoding sequences whose common ancestry with the TRGF preceded the origin of the TRGF. In the process, we were able to confirm the recent de novo status of the first, and refute the apparent taxonomic restriction of the second.

The first TRGF evolved de novo in the *simulans–sechellia* clade on chromosome 3 R, giving rise to *Dsim_GD19764* and *Dsec_GM10790*. These are annotated uncharacterized protein-coding genes with two CDSs and a conserved canonical GU–AG splicing signal. The protein is 129 amino acids long in *D. simulans* and 113 in *D. sechellia*. The conserved intron is 52 nucleotides long (not a multiple of 3), hence it is likely to predate the ORF (otherwise, later intronization would have resulted in a frame-shift; see Yang and Huang [2011] for a detailed explanation). BUSCA predicts that this TRGF contains a transmembrane alpha helix and hence localizes to the endomembrane system. We checked *Dsim_GD19764* and *Dsec_GM10790* for presence of known protein domains, but found no hits. TANGO predicts that *Dsim_GD19764* and *Dsec_GM10790* have no regions prone to aggregation. There is transcriptomic evidence that *Dsim_GD19764* is expressed in the male reproductive system (Drosophila 12 Genomes Consortium 2007), which is in line with previous results showing that TRGs are predominantly expressed in testes (Levine et al. 2006).

**Fig. 4.**
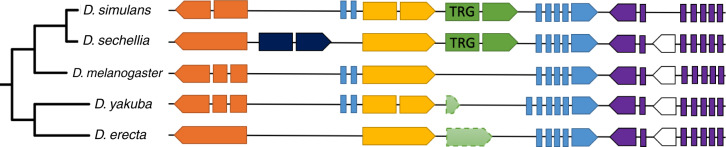
DNA regions homologous to the TRGF containing *Dsim_GD19764* and *Dsec_GM10790*. Homologous protein-coding genes are the same color (each element corresponds to an exon), small nuclear RNA (snRNA) genes are white. The direction of the arrow shows which strand the gene is located on. Features with dashed outlines are not annotated. The diagram is not to scale. In the order from top to bottom, the orange genes are *Dsim_GD29138*, *Dsec_GM10660*, *Dmel_CG12589*, *Dyak_GE25310*, *Dere_GG11200* (with a syntenic homolog *Dana_GF16073* in *Drosophila ananassae*); the yellow genes are *Dsim_GD19763*, *Dsec_GM10789*, *Dmel_CG12590*, *Dyak_GE25451*, *Dere_GG12627* (with syntenic homologs *Dana_GF18925* and *Dpse_GA11706* in *D. ananassae* and *D. pseudoobscura*, respectively); the blue genes are *Dsim_GD19765*, *Dsec_GM10791*, *Dmel_CG12591*, *Dyak_GE25452*, *Dere_GG12638* (with syntenic homologs *Dana_GF18926* and *Dpse_GA11707* in *D. ananassae* and *D. pseudoobscura*, respectively); the purple genes are *Dsim_GD19639*, *Dsec_GM10658*, *Dmel_CG12161*, *Dyak_GE25306*, *Dere_GG11178*.

*Dsim_GD19764* is located in an intron of a conserved protein-coding gene *Dsim_GD19765*, downstream of conserved protein-coding gene *Dsim_GD19763* located inside the same intronic region (fig. 4). In *D. sechellia*, the *Dsec_GM10791* gene harboring two genes inside its intron appears to have lost the first two exons, and thus *Dsec_GM10790* is located in a similar genomic context but not inside an intron. The DNA regions that we presume to be homologous to TRGs in *D. melanogaster*, *D. yakuba*, and *D. erecta* are located between the genes homologous to the ones neighboring TRGs in the *simulans–sechellia* clade. There is too little nucleotide conservation for a good alignment to this region in *D. melanogaster*, which contains no ORF. Alignment can be achieved with the *yakuba–erecta* clade, where the ORF is disrupted by an early stop codon in *D. yakuba* and several indels including an early frameshift plus loss of splicing signal in *D. erecta*.

Note that Hild et al. (2003) previously inferred a protein-coding gene in *D. melanogaster* located in this region on the opposite strand, but this gene is no longer part of the official genome annotations. Because of this “homologous” hit, Heames et al. (2020) classified this TRGF as originating through rapid divergence rather than de novo. However, even if this no longer annotated sequence did encode a functional protein, the fact that it is on the opposite strand means that it should not be classified as a diverged homolog. De novo origination that occurs in alternative reading frames is still de novo origination.

We identified homologous DNA regions in four additional outgroup species (*Drosophila ananassae*, *Drosophila suzukii*, *Drosophila pseudoobscura*, and *Drosophila miranda*), and although the sequence conservation level was insufficient to provide precise information about the most likely ancestral state, no start codon was present in these homologous DNA regions. We can thus rule out the possibility that two independent pseudogenization events, one in *D. melanogaster* and one in the basal lineage of the *D. yakuba–erecta* clade, created the illusion of a TRGF as a false-positive. The homologous regions in *D. ananassae* and *D. pseudoobscura* contain three (orange, yellow, and blue in fig. 4) and two (yellow and blue in fig. 4) syntenic homologs, respectively, whereas the putative homologous regions in *D. suzukii* and *D. miranda* (identified via BlastN alone) contain none. Using protein sequences of the TRGF to perform TBlastN search resulted in partial hits (covering 45–58% of the sequence) with 47–53% sequence similarity in *Drosophila eugracilis, Drosophila ficusphila, Drosophila rhopaloa, Drosophila elegans*, and *Drosophila biarmipes*. Due to the lack of syntenic evidence, we were unable to confirm whether these are truly homologous regions, but absence of hits from the syntenic region in closer relatives makes this unlikely. It was not possible to obtain an informative multiple sequence alignment of these highly diverged sequences, and hence no additional information was acquired from these hits.

The second gene family, that our pipeline mistakenly identified as a TRGF, contains uncharacterized protein-coding genes *Dsim_GD20667* and *Dsec_GM19408*, and an unannotated homologous ORF in *D. melanogaster*. These annotated genes are located on the 3R chromosome and contain a single CDS of length 155 in *D. simulans* and *D. melanogaster*. In *D. sechellia*, a frameshift close to the end of the CDS results in a conserved stop codon becoming in-frame and thus shortening the CDS to 139 amino acids. BUSCA predicts that the proteins localize in the nucleus.

**Fig. 5.**
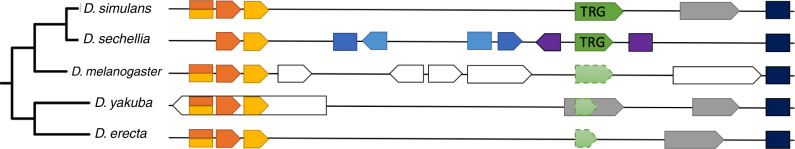
DNA regions homologous to the gene family containing *Dsim_GD20667* and *Dsec_GM19408*. Protein-coding genes are shown in color, pseudogenes in gray and ncRNA genes in white. Homologous protein-coding genes are marked by the same color, each element corresponds to an exon. Only the first of the seven exons of the dark blue gene is shown. Genes shown directly above/below each other share sequence similarity, but we did not infer homology of all pseudogenes and ncRNA in a rigorous way. The direction of the arrow shows which strand the gene is located on. Features with dashed outlines are not annotated. The diagram is not to scale.

These putative TRGs are located among protein-coding gene families syntenically conserved in all five subgroup species, ∼70 kb downstream from a conserved pair of overlapping genes and ∼25 kb upstream from a conserved seven exon gene. The region between these two gene families is shown in figure 5.

A number of protein-coding genes are annotated in *D. sechellia* but have no detectable homologs in other species in the subgroup. *Drosophila melanogaster* has a number of annotated ncRNAs, one of which overlaps with parts of *D. sechellia*-specific genes. Since these protein-coding genes are present in only one species, we did not include them in our analysis, because in the absence of conservation, we lack sufficient evidence that they are functional. The region containing the putative TRG is annotated as an intron of one of these *D. sechellia* protein-coding genes. The downstream region annotated as a pseudogene in *D. simulans*, *D. yakuba*, and *D. erecta*, and as a ncRNA in *D. melanogaster*, is well-conserved in all species. The annotation boundaries vary among species.

The region containing the TRGF is extremely well-conserved in all five species and is annotated as a pseudogene in *D. yakuba*. Using BlastN for similarity searches to identify the parent gene of this putative pseudogene, we were only able to find a self-hit and numerous matches covering <10% of the sequence in all species with an exception of *D. simulans* where we identified a 219 nucleotide long unannotated contig with 97.7% sequence identity. We were unable to find any other evidence about the parent gene of this putative pseudogene, casting doubt on its pseudogenic nature.

**Fig. 6.**
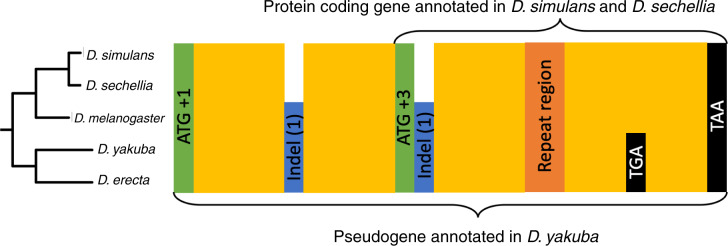
Sequence features of the ancestral ORF, which is annotated as a pseudogene in *Drosophila yakuba*. Start codons of the annotated pseudogene and of the shorter putative TRG in the *simulans–sechellia–melanogaster* clade are in green, well-conserved regions in yellow, frame-shift causing indels in blue, repetitive DNA in orange, and stop codons in black. We use the following frame numbering convention: the start codon of the putative pseudogene is denoted the +1 frame, the other two frames on the same strand are denoted +2 and +3 frames. The numbers in parentheses indicate how many more nucleotides (modulo 3) the species it is marked in has. The frames of the stop codons are not marked due to uncertainty about frame created by the repeat region. The two stop codons shown are located in the same frame.

The start codon of the putative TRGF is in a different frame than that of the *D. yakuba* putative pseudogene, suggesting that it evolved de novo in an alternative frame, but upon closer scrutiny, we realized that this is not the case. The start codon of the putative TRGF is flanked by two indels, which brings the frame of the annotated *D. yakuba* pseudogene in frame with the putative TRGF following its annotated start codon (see fig. 6). A *TG*-dinucleotide repeat region in the middle of the putative TRGF ORF appears to be poorly conserved; this could be either because of a genuinely higher mutation/indel rate, or merely because of a poor quality of reads/assembly in this region. The uncertainty created by this region and the fact that the length of the pseudogene is not a multiple of three makes it difficult to infer whether the putative TRGF shares the frame with the pseudogene throughout the whole sequence.

More telling information comes from six stop codons conserved across the five species and located between the repeat region and the stop codon shared by both the pseudogene and the putative TRGF. Four stop codons are in +2 frame of the putative TRGF and 2 stop codons are in the +3 frame, leaving +1 as the only frame of the putative TRGF free from stop codons conserved across the five species. If we assume that the pseudogene was free from stop codons when it originated, then this implies that the putative TRGF sequence following the repeat region is in the same frame as the original pseudogene sequence.

The fact that we were unable to identify the ancestral gene associated with this pseudogene, the high level of sequence conservation (95% sequence identity between *D. yakuba* and *D. simulans*, excluding 25 out of 587 nucleotides corresponding to indels), and more than 110 amino acid long ORF still present in *D. yakuba* all add up to substantial evidence that this *D. yakuba* ORF annotated as a pseudogene might in fact be a misannotated functional gene. Regardless of whether this pseudogene is a true remnant of a previously functional gene or a misannotated gene that is still functional today, we conclude that the putative TRGF in *simulans–sechellia* did not evolve de novo. Instead, it evolved from an ancestral protein encompassing all yellow regions shown in figure 6, via truncation of the N-terminal, whose homologous sequence does not appear to be in the same frame as it is in the rest of the clade.

## Discussion

The aim of this study was to identify high-confidence TRGFs as the most promising candidates for experimental studies of protein-coding genes that emerged in the past 3.3 Myr, while avoiding ascertainment biases associated with preconceptions of how de novo genes are born. We will only learn about how de novo genes are different from well-established genes if we look for them with an open mind and without assumptions that their sequences must be similar to well-established genes in order to be functional. Unlike other studies, we did not filter out genes with composition distinct from average composition of sequences in protein databases (Vakirlis et al. 2018) nor assume that TRGFs cannot contain splicing signals (Knowles and McLysaght 2009). To avoid including candidates that are not functional protein-coding genes, without making such assumptions, we used ORF conservation as a proxy for selection and hence evidence for functionality, in addition to using NCBI genome annotations as the most comprehensive synthesis of evidence for transcription and/or translation. To avoid including candidates that were not born de novo, we conducted extensive investigation of homologous noncoding sequences in sister species.

We identified a single TRGF with annotated single-copy genes in *D. simulans* and *D. sechellia*. This TRGF is located in a syntenic context conserved across all examined species in *D. melanogaster* subgroup. It contains an intron that predates birth as an ORF. Due to the lack of sequence conservation outside the *D. melanogaster* subgroup, we were unable to establish whether enabling mutations (indels and substitutions) occurred after divergence of the *simulans–sechellia–melanogaster* clade, or earlier followed by loss in the common ancestor of *D. yakuba* and *D. erecta*. Our results highlight that de novo gene studies should under no circumstances exclude candidate TRGs just because they have introns.

The number of de novo genes reported in any study depends on the balance of false-positives and false-negatives that has been achieved by the authors. This is shaped by decisions as to what counts as evidence for functionality and what properties of the candidate genes signify that they are not true de novo genes. When we began this study, our requirement that a de novo gene must have homologous noncoding DNA sequence(s) in outgroup species as evidence for the time of emergence was stricter than most. Since then two papers have been published that described (Vakirlis and McLysaght 2018) and applied (Zhang et al. 2019) a similar requirement for homologous noncoding DNA sequences in outgroup species.

Zhang et al. (2019) examined de novo genes in the *Oryza* clade and concluded that about 51.5 de novo genes per million years are generated and retained in this clade. Although care was taken to show de novo status, this number is nevertheless likely inflated by lenient criteria for functionality. Intact gene structure and some transcription and translation were considered sufficient, with no requirement for functional evidence or evolutionary conservation. The estimated rate of de novo gene birth is also potentially deflated (but not by as much) by the assumption that recent de novo genes cannot be present in more than one copy. Another limitation of the study is that only the single best hit to a genome was considered. Since hits were accepted if they covered ≥20% of an ORF, this could lead to selecting a short highly similar region (e.g., to a low complexity region) and ignoring a longer truly homologous region with a slightly lower match score. Accepting matches that cover as little as 20% of an ORF is contradictory to the idea presented in the paper that indels and substitutions are the main ORF triggers, and may have deflated the estimate.

In contrast, our study, which was designed to identify high-confidence experimental candidates, is likely an underestimate, in part because homologous sequence in orthologs might be missing or unrecognizable, but mostly because it cannot find a TRGF unless it is already present in the NCBI gene annotations of two species. The incomplete nature of genome annotation is more of a problem when a gene must be annotated in two species than when it must merely be annotated in one. Abascal et al. (2018) shows that ∼12% of human genes have different annotations across the three most popular databases (RefSeq, Ensembl/GENCODE, and UniProtKB), and that some genes that are listed as noncoding actually have more experimental evidence for producing a protein than some genes listed as protein-coding. Even in relatively simple species like *E. coli*, ∼35% of the annotated genes lack experimental evidence of function (Ghatak et al. 2019). The annotation quality for the *D. melanogaster* subgroup is unlikely to be better than for the human genome. Nevertheless, we believe that synthesis of evidence from all data sets submitted to NCBI is by far better than the evidence that we could have gathered and synthesized ourselves without performing experimental work.

The availability of evidence for functionality is the limiting factor in identifying very young genes. Without it, short young proteins are often left out of genome annotations, and hence alternative approaches like screening all ORFs present in a genome (Ruiz-Orera et al. 2018) are required to identify them. Given the frequency of premature stop codon mutations, conservation of an ORF across several species can be used as a proxy for functionality, as we do here. However, sufficiently short ORFs can still be conserved by chance sometimes across several species.

One reason we find a lower rate of de novo gene birth might be that false-positive evidence of functionality inflates single-species estimates in other studies, whereas false-negative failure to reproduce such evidence in two species deflates it in our study. However, it is also possible that both estimates are approximately correct, with the discrepancy arising from the fact that rapid emergence of functional ORFs is counter-balanced by rapid loss, as discussed by Schlötterer (2015). Since newborn proteins are not yet integrated in the protein interaction network, they might be relatively dispensable; even if adaptive at first, they might not remain adaptive as the environmental and genetic context changes. In this case our approach, in using evolutionary conservation to exclude nonfunctional polypeptides, also excludes functional proteins whose functionality is short-lived.

There have been several previous papers aimed at identifying TRGs in *D. melanogaster* subgroup: the pioneering work of Levine et al. (2006) focused on de novo genes, followed by a survey of all TRGs (Zhou et al. 2008), a study about essentiality of TRGs (Chen et al. 2010), an in-depth analysis of the evolution and function of six candidate de novo genes (Reinhardt et al. 2013), and a study of very young de novo genes in *D. melanogaster* that are still segregating in the population (Zhao et al. 2014). These studies collectively reported 16 de novo protein-coding genes and two de novo ncRNAs (Dme_CR32582, Dmel_CR32690) that are fixed in *D. melanogaster* and not present outside of the *D. melanogaster* subgroup. Three of these protein-coding genes (Dmel_CG33235, Dmel_CG33666, Dmel_CG34434) are present only in *D. melanogaster* and hence were not included in our analysis, and another seven of them (Dmel_CG2042, Dmel_CG32582, Dmel_CG32690, Dmel_CG32824, Dmel_CG40384, Dmel_CG9284, Dmel_CG32582) have been removed from the genome annotations since the time of publication. For the remaining six previously reported de novo protein-coding genes, we were either able to identify homologous genes outside the *D. melanogaster* subgroup (Dmel_CG31882, Dmel_CG30395, Dmel_CG31406, Dmel_CG32712), or we were unable to identify homologous DNA regions in any of the outgroup species (Dmel_CG15323, Dmel_CG31909). Note that these last two could still be de novo genes. Here, we have identified a TRGF containing *Dsim_GD19764* and *Dsec_GM10790* in *D. simulans* and *D. sechellia*, respectively, that evolved de novo. This TRGF is not present in *D. melanogaster* and hence was not part of these previous studies. We did not identify any TRGFs in this clade that evolved de novo and contain an annotated *D. melanogaster* gene.

The most recent study by Heames et al. (2020) identified 32 putative de novo TRGFs in *simulans–sechellia–melanogaster* clade. None of these 32 was supported by our analysis. For 25 of them, we identified BlastP hits outside the *D. melanogaster* subgroup, and for 3 of them, we identified conserved but unannotated ORFs in outgroup species. This indicates an earlier origin of these TRGs, as well as emphasizing the importance of these two quality control steps. For 2 of them (one consisting of FBgn0269617 in *D. simulans* and FBgn0169891 in *D. sechellia*, the other consisting of FBgn0268387 in *D. simulans* and FBgn0168374 in *D. sechellia*), the candidate gene in *D. sechellia* was no longer part of the official genome annotations (meaning that we failed to get our minimum of two annotated homologs). Although these two might still be genuine TRGFs, we note that poorly assembled genomes contain more spurious genes, and that this is reflected in the relative numbers of singleton TRGs reported by Heames et al. (2020), with 41 in *D. melanogaster*, 251 in *D. simulans*, and 958 in *D. sechellia*. For the remaining two putative de novo TRGFs, the two homologs did not meet our length tolerance ratio of 61% of aligned homology: length of shorter protein (see Materials and Methods), so that our pipeline did not infer them to be homologous. The gene pair of FBgn0268561 in *D. simulans* and FBgn0266534 in *D. melanogaster* had a ratio of 43.06%, whereas the gene pair of FBgn0269153 in *D. simulans* and FBgn0267104 in *D. melanogaster* had a ratio of 54.17%. As discussed earlier, the one TRGF that we did identify with high confidence was not found by Heames et al. (2020) because a homologous nucleotide sequence used to have a protein-coding gene annotated on the opposite strand, and this was taken by Heames et al. (2020) to be evidence of origination by divergence instead of de novo.

Our results show that although de novo genes that are conserved across several species undoubtedly do exist, their number is probably on the lower side of the spectrum of estimates reported in previous studies. We have identified only a single TRGF in the *D. melanogaster* subgroup, which does not allow us to identify a common pattern of emergence of de novo genes. High confidence in its annotation as de novo and as conserved may make this de novo gene the best candidates in *D. melanogaster* subgroup identified so far for the experimental studies needed to drive the field forward.

## Materials and Methods

### Data

The genome assemblies for *D. melanogaster*, *D. simulans*, *D. sechellia*, *D. yakuba*, and *D. erecta* were downloaded from RefSeq (Haft et al. 2018) along with the genome annotations (O’Leary et al. 2016). The completeness of the protein sets was assessed using BUSCO (Waterhouse et al. 2018), using 2799 Hidden Markov Models of single-copy orthologs found in >90% of species in the order Diptera. Table 1 summarizes genome statistics for each species.

### Homology Predictions

We used OMA v2.2.0 implementation of the OMA algorithm (Altenhoff et al. 2018) with default parameters to infer groups of homologous genes across six genomes: five *D. melanogaster* subgroup species and *D. ananassae* as an outgroup. The length tolerance ratio was set to the default value of 61%, meaning that if the length of the alignment between a putative pair of homologous proteins is <61% of the length of the shortest of the two proteins, then no homology was inferred. All the genes annotated as protein-coding in the assemblies described above were used, regardless of their length. We selected orthologous families with genes in at least two of the species in the *simulans–sechellia–melanogaster* clade and no genes outside this clade as putative TRGFs for further analysis.

### Validation of Putative TRGFs

Putative TRGFs were first validated with sequence similarity searches in amino acid space against all nonredundant proteins in the RefSeq database, using BlastP v2.7.1+ (Camacho et al. 2009) with default parameters. All hits with e-value ≤1e-03 and covering ≥50% of the query were considered. If every gene in a putative TRGF had at least one hit to the species outside the clade, the family was removed from further validation. We did not try to identify highly diverged homologs that are beyond detectability with BlastP using more advanced methods like PSI-BLAST (Schaffer 2001), HHMER (Eddy 2011), or HHblits (Remmert et al. 2012) that rely on building a sequence profile. There were two reasons for this. First, given that the protein is only present in two species the resulting sequence profile would not contain much more information than a single sequence and hence it would be unlikely to yield useful results. Second, we relied on our assumption that if a homologous gene is present in an outgroup genome it would be included in the BlastN hits against that genome. This assumption does not necessarily hold at large evolutionary distances, but for closely related species it would be extremely unlikely to identify a good DNA sequence match covering all of the gene and at the same time to miss a homologous gene that diverged beyond detectable similarity in nucleotide sequence space.

Remaining putative TRGFs were validated with sequence similarity searches in nucleotide space against the five genomes in *D. melanogaster* subgroup, using BlastN v2.7.1+ (Camacho et al. 2009) with default parameters. We did not use tools like FASTA3 (Pearson 2000) that take into account synonymous codons or amino acid similarity because the homologous DNA sequences are protein-coding in some species but not the others. BlastN makes no additional assumptions about the evolutionary constraints specific to the query sequence, and hence is most suitable tool for this problem. For each gene, we used both the whole gene sequence and the set of CDSs as a query. This approach ensures that hits to even very short CDSs are retained, while also using the information in the noncoding parts of the gene when the information contained in a short CDS is insufficient. All hits with e-value ≤1e-03 and covering ≥50% of the query (a whole gene or a CDS) were considered, and overlapping hits were amalgamated. In cases where the total number of hits exceeded 1,000, we ordered the hits by e-value and selected the five best hits per species. Hits (including self-hits to the genes) were aligned with MAFFT v7.407 (Katoh and Standley 2013) using E-INS-i algorithm that makes minimal assumptions about the nature of the resulting alignment. We used the “–adjustdirectionaccurately” option to align hits located on different strands and the “–addfragments” option to subsequently add CDSs to the alignment of hits. Alignments were examined manually to remove the hits that were only covering parts of introns or untranslated regions and to extend promising hits that ended in the middle of the gene. After these amendments the remaining/extended hits were realigned and the resulting alignments were examined for presence of homologous ORFs in the *yakuba–erecta* clade. If an ORF was identified in at least one of the two outgroup species, it was considered as evidence that the putative TRGF originated prior to the speciation of the *D. melanogaster* subgroup and the family was removed from further validation. Putative TRGFs that passed sequence similarity validations were manually examined for quality and consistency of annotations.

### Inferring the Origin of TRGFs

To infer the origins of TRGFs, we extracted genome annotations corresponding to the identified homologous DNA regions in all five species. Synteny conservation in these DNA regions was used as evidence of homology for the less conserved sequences. We also identified homologous DNA regions in four additional species—two in the *melanogaster group* (*D. ananassae* and *D. suzukii*) and two in its sister clade *obscura group* (*D. pseudoobscura* and *D. miranda*). We checked for presence of known protein domains with HMMER v3.1b2 (Eddy 2011) using Pfam v31 database (Finn et al. 2016). We used the BUSCA web server to predict protein subcellular localization (Savojardo et al. 2018), TANGO to predict protein aggregation (Fernandez-Escamilla et al. 2004), and Wasabi for visualizing multiple sequence alignments (Veidenberg et al. 2016). All analysis was performed in Python v3.7.0, using packages biopython v1.73 (Cock et al. 2009) and gffutils v0.9. The code is available at https://github.com/KarinaZile/TRGs_in_Drosophila_melanogaster_subgroup (last accessed July 15, 2020). 

## Supplementary Material

evaa127_Supplementary_Data
