# Supplementary material for: Only a Single Taxonomically Restricted Gene Family in the Drosophila melanogaster Subgroup Can Be Identified with High Confidence
Source: Genome Biol Evol. 2020 Jun 26;12(8):1355–66. doi: 10.1093/gbe/evaa127 (PMC8059200; doi:10.1093/gbe/evaa127)
Supplement: evaa127_Supplementary_Data [file evaa127_supplementary_data.zip › supplementary_material.pdf]

Supplementary materials

TRGF 1 sequences

```
>Dsim_GD19764
ATGTGGCAGGCCACATCCTCCTCGTTTTCCAGCAAGCCGCCAAGGAGCGAGAAATTCCGGAG
CAGCTGCTCCAACTACAGTGTCATCTTGAATCACAACTATGCCGGCCGGCAAGATGACCGCA
AAATTTGTGAACAGTCGGACCAAGACCAGGCCACACCCCAACAGCTGAACCACCGCAAGGGC
GGCGGAGCTCTGGAGTTGGTCCTGCAGAAAGGCCAGCTGTTCCACCCACTGGAGCAACATGC
CCAGCGCGTCCTCGCACCACCATGGGCAGACCGGATGACCTTTTGCCCAGATCCACCGCCCG
TTCTGAAAAGGGGGTCCCCTACTGCGTCGGGAGCATGTTCCGAGATTCCTGCTCCAAGCCGC
AAGACTACTGCGAGCTAA
>Dsec_GM10790
ATGTGGCACACATCCTCCTCGTTTTCCAGCAAGCCGCCAAGGAGCGAGAAATTCCGGAGCAG
CTGCTCCAACTACAGTGTCACTTTGAATCACAACTATGCCGGCCGGCAAGATGACCGCAAAA
TTAGTGAACAGTCGGACCAAGACCAGGCCACAACCCAACAGCTGAACCACCAGATGATTGAG
CAGGACTACAGCCACCGCAAGGGCGGCGGAGCTCTGGAGTTGGTCCTGCAGAAAGGCCAGCT
GTTCCACCCACTGGAGCCACATGCGCTGCGCGTCCTCGCACCACCATGGGCAGACCGGATGA
CCTTTTGCCCAGATCCAACGCCCGTTCTGTAA
```

TRGF 1 conserved region alignment

```
>DROSI-NT_479536.1-896197:897745
CGGGGAGGCGTCCCCCGAAAGTAGGCAATCCACGGATGGGGGTGGATGGGTGCTTGGGTG
CCT-GGTGACCCCAGCCCACAGTGGGAGTACAATGTGGGCGGCATGACTCGGTAATTCCA
GGCCCATCTGCGGCATTTTATGCTCCAATAAAATCGCAATGTGGCCGCGAGCTTAAAGCT
TGATTGGATCAGGAAAGGGACGTGGAACAGTGACCAGTCCACCATTGTTTCATTAGGTGC
TCCCAGAAGAGTCAAGTACAGAGAATTTGCCAAAGAATGACGACAATCTGTGCTTGGGAG
ATTGAAACACTGTTCATTATAAGATCCCTTCTAGCTGTTCGCCCCATAAACCATCCGAAA
ATCTAGGGAGTTACAGAGCCTTCTTTAACGCATAAACTGAAAAGGCATATATCAACTGAT
GAACAGGACTAATAATCTTAATTAGGAATGAAGTGGACACCACTGACGATAACTGCAAAA
AGTTGTACTTCAGCGATCTGCTGTGCAGCTCTATCGCGATGGATGGGCGAATTGGTGGAC
ACATTACTCATGTTCAGAACAAAGACATTATAATGTATAGAATATTCTGTCTTCGGTCAG
AATAAATGGTGTATTAGCCCACTACGACATAGAATATAAGGAAGATACCAAAGCAGGTCA
GGACTCTAGTGCTGGAACACGGTCAGAGGAAATACAGCTCCTGCTCCGT-GATGTGGCAG
GCCACATCCTCCT------CGTTTTCCA-----------GCAAGCCGCCAAGGAGCGAGA
AATTCCGGAGCAGCTGCTCCAACTACAGTGTCATCTTGAATGTGGGAATCTGAGTAATAC
AC-TAAATTT-----------------AATAAGACAGGCTG-------------------
GGCTAACGACTCCAAATTTACCTGAAGCACAACTATG-------CCGGCCGGCAAGATGA
CCGCAAAATTTGTGAACAGTCGGACCAAGACCAGGCCACACCCCAACAGCTGAA------
---------------------CCACCGCAAGGGCGGCGGAGCTCTGGAGTTGGTCCTG--
CAGAAAGGCCAGCTGTTCCACCCACTGGAGCAACATGCCCAGCGCGTCCTCGCA------
--CCACCA------------------TGGGCAG------------ACCGGATGACCTTTT
GC-CCAGATCCACCGCCCGTTCTGAAAAGGGGGTCCCCTACTGCGTCGGGAGCATGTTCC
GAGATTCCTGCTCCAAGCCGCAAGACTACTGCGAGCTAAGAGGATGACAGTCGTGGCAGT
GATAACAAACGGCGTACGCCAGGCTGAGTAATCGGTCCGGTCTGCCTTTAGTCTGTGGCA
CATCCTGGAGAGATTGCTTCGCTACGAGGCGTCGTAAGAGGTCCTTTTCCGGGCACAGCT
GTCGTTTCGGAAATTTCATTTCGTAATGCGCTCCTGTCCTATTAACATACATATTGTACC
ATCTTCGTAGAAAAACTGATTTAGATGAATTATATTTAAAAGAATTTTTTTAAATATGAA
ATTTGTAGCCTAAGCTAACTTGAAATTGGTCCGGAATCCCAGTCATCCGATGGCATCACT
```

GCTTTTGAAGAGCGTTTCTCTACACAGATAACCATCAAAATATATCCCAAAATGAACCTA
>DROSE-NW_001999695.1-1113339:1114909
CGGGGAGGCGTCCCCCGAAAGTAGGCAATCCACGGATGGGGGTGGATGGGTGCTTGGGTG
CCTGGGTGACCCCAGCCCACAGTGGGAGTACAATGTGGGCGGCATGACTCGGTAATTCCA
GG-CCATCTGCGGCATTTTATGCTCCAATAAAATCGCAATGTGGCCGCGAGCTTAAAGCT
TGATTGGATCAGGAAAGGGACGTGGAACAGTGACCAGTCCACCATTGTTTCATTAGGTGC
TCCCAAAAGAGTCAAGTACAGAGAATTTGCCAAAGAATGACGACAATCTGTGCTTGGGAG
ATTGAAACACTGTTCATTATAAGATCCCTTCTAGCTGTTCGCCCCATAAACCATCAGAAA
ATCTAGGGAGTTACAGA-CCTTCTTTAACGCATAAACTGAAAAGACATATATCAATTGAT
GAAAAGGACTAATAATCTTAATTAGGAATGAAGTGGACACCACTGACGATAACTGGAAAA
AGTTGTACTTCAGCGATCTTCTGTGCAGCTCTATCGCGATGGATGGGCGAATTGGTGGAC
ACATTACACATGGTCAGAACAAAGACATTATAATGTATAGAATATTCTGTCTTCAGTTAG
AATAAACGGTGTATTAGCCCACTACGACATAGAACATAAGGACGATACCAAAGCAGGTCA
GGACTCTAGTGCTGGAACACGGTCAGAGGAAATACAGCTCCTGCTCCGT-GATGTGGCA-
--CACATCCTCCT------CGTTTTCCA-----------GCAAGCCGCCAAGGAGCGAGA
AATTCCGGAGCAGCTGCTCCAACTACAGTGTCACTTTGAATGTGGGAATCTGAGTAAATC
AC-TAAATTT-----------------AATAAGACAGGCTG-------------------
GGCTAACGACTCCAAATTTACCTGAAGCACAACTATG-------CCGGCCGGCAAGATGA
CCGCAAAATTAGTGAACAGTCGGACCAAGACCAGGCCACAACCCAACAGCTGAACCACCA
GATGATTGAGCAGGACTACAGCCACCGCAAGGGCGGCGGAGCTCTGGAGTTGGTCCTG--
CAGAAAGGCCAGCTGTTCCACCCACTGGAGCCACATGCGCTGCGCGTCCTCGCA------
--CCACCA------------------TGGGCAG------------ACCGGATGACCTTTT
GC-CCAGATCCAACGCCCGTTCTGTAAAGGGGGTCCCCTACTGCGTCAGGAGCATGTTCC
GAGATTCCTGCTCCAAGCCGCAAGACTACTGCGAGCTAAGAGGATGACAGTCGTGGCAGT
GATAACAAACGGCGTACGCCAGGCTGAGTAATCGGTCCGGTCTG-CTTTAGTCTGTGGCA
CATCGTGGAGAGATTGCTTCGCTACGAGGCGTCGTAATAGGTCCTTTTCCGGGCACAGCT
GTCGTTTCGGAAATTTCATTTCGTAATGCGCTCCTGTCCTATTAACATACATATTGTACC
ATCTTCGTAGAAAAACTGATTTAGATGAATTATATGTAAAAGAATTTTTTTAAATATGAA
GTTTGTAGCCTAAGCTAAATTGCAATTGGTCCGGAATCCCAGTCATCCGATGGCATCACT
GCTTTTGAAGAGCGTTTCTCTAAACAGATAACCATCAAAATATATCCCAAAATGAACTTA
>DROME-NT_033777.3-5163432:5164465

---------TCCATGTCCGCAA------------GTTTGCAGGAAAATTAAGTTACTTAA
AG-CCAACTCCAGCGGCT--
-------------------ACTTAGGCGGAGGAACTGCCTGCAGCTGTGCTG--------
-CTCGCAAGTGGAAAGTGCAAAG----TGGAAAACTATTTTTGCAAATTGTTCTCGGGCG
A---
--AGCGTTTAAT
AAAAA---
-----------TGTAAACTTGAGTGCAACTCCACTGCAGT----------CTCTGCTGAC
TTTATGCTTGTATTTGGCA---

-------------TAATATTATTTACATAATAAATACTCATTTGTTGTTTGTTGTGCTGC
GCGTAAATTTGCCGCCCGAGGCGCGACACTGGAACGGGGCGGCGTCTCCCGAAAGTAGGC
AATCCACGGATGGAGGTGGAGGTGGGAGTAGAATGTGGTCATACCGAGCCAGCAAGGTGA
AAGCAAAATTTGTGAACAGTCGGACCAAGACCAGACCACAGCCCAACAGCTGAACCACCA
GATG---GAGCAGGACTACGGCCACCGCATGGGTGGCGGAGCTCAGGAGCTGGTCCTG--
CAGAAAGGCCAACTGTTCCACCCACTGGAGCCACATGCCCAGCGCGTCCTCGCA------
--CCACCA------------------TGGGCAG------------ACCGGGTGACCTCTT
GCCCCAGATCCA-------TTCTGGAAAGGGGGTCTCCTTCTGCGTCAGAAGCATGTTCT

```
GAGATTCCTGCTCCAAGCCACAAGACTACTGCGAGCTAAGAGGATGACAGTCCTGGCAGT
GATAACAAACGGCGCACGCCAGGCTGAGTAATCGGTCCGGTCTGCCCTTAGTCTGTGGCA
CATCCTGGAAAGATGGCTGTGCTACTAGGCGTCGTAAGAGGTCCTTTTCCGGGCACAGCT
GTCGTTTCGGGAATTTCATTTCGTAATGCGCTCCTGTCTTATTAACATACATATTTTACC
ATCATAGTAGGAGAACTGGTTTAGAAGAATTGTATTTAAACGAATTTTTTTAAATGTGTA
ATTTGTAGCTAAG------------------CGGAGTCCATATCATCTGATGGCATAACT
GCTTTTGATGGGCGTTTCTCTACACAGATAACCATCAAAATATATCTCAAATTGAA----
>DROYA-NT_167065.1-1351799:1352543
------------------------------------------------------------
------------------------------------------------------------
------------------------------------------------------------
------------------------------------------------------------
------------------------------------------------------------
------------------------------------------------------------
------------------------------------------------------------
------------------------------------------------------------
------------------------------------------------------------
------------------------------------------------------------
------------------------------------------------------------
------------------------------------GCTCCTGCTCCGAAGATGCGGCAG
GCCTAAGCCTCCT------TGTCCTCCACATGCAGGAGCAGAAGGAGCCAAGGAGCGA--
-------------TAGTTTTAGCTGCAGTGTCACTTCGTATCTGTTAGCCTGTGTAATAC
---TAAATTT-----------------AATGGGGCAGGCTG-------------------
AGCTCACGACTCCAAACTTAACTGAAGCAGAACTACG-----------------------
-----AGTATACCGGCCTGTAGGAGTAGGACCAGACC-----------GCTGACCCAACA
TATT---GAGCAGGACTACTGCCGC--------CGGTGGAGCACTGGATCTGGTCCTG--
CAGAAAGACCAGCTGTTCCACCCACTGGA-CCACATGCGCAGCGCGCCTTCGAAGAAGGT
GGCCACCAGGCAGAGATGAAACACCATGAGCGGGGAGACGGCCCCGCCGGTTGACTTGCC
---CCATATCCACCGCCCTTTCTA-GAATGGGGTCCCAGCCTGCGTCAGGAGCATGCTCT
CAGATTCCGACTCCAAGCCGCAAGTTCACTTTAAGCTATGAGTATGACAGTCGTGGCAGT
GATAACAAACGGCCCACGCCAGGCTGAGTAATCGGTACGGTCTGCCTCTAGTTTGTGGCA
CATCCTGAAAAGATTGCTGCGTTACGAGCCGTCCTAAGTAGTCCTTTTCCGGGCACAGCT
GTCGTTTCGGGAATTTCACTTCGTAATGCGTTCCTGCCCTATTAACACACATATTTTATC
AT------AGAAAAAATGGGTTATTAGTATTGTATTTAAACGAACATTTTTTAATATGAA
ATTT--------------------------------------------------------
------------------------------------------------------------
>DROER-NW_001956552.1-1266146:1266887
------------------------------------------------------------
------------------------------------------------------------
------------------------------------------------------------
------------------------------------------------------------
------------------------------------------------------------
------------------------------------------------------------
------------------------------------------------------------
------------------------------------------------------------
------------------------------------------------------------
------------------------------------------------------------
------------------------------------------------------------
------------------------------------GCTCCTGTCCCGTGGATGCGGCAG
GCCACAGCCTCCTTGTCCCTGTCCTCCACATGCAGGAGCAGCAACCCGCAAGGAGCGAGA
AATTCC-AAGCAGCAGCTTCAGCTGCAGTGTTACTTCGCATCTGTGAGCCTGAGTAATAC
---TAACTTT-----------------GTTAA----------------------------
```

```
----------TCCAAATTTACCTGGAGAAGCACTATG-------CGGGCCAGCAAGGTGA
CCGCAAAATTGGTGAACAGTCGGACTGAGACCAGGCCACAGTCCAGCAGCTGACCAACCA
AAAT---GAGCAGGACTATGGCCACCACAAGGCCGGTGAAGCACTGGAGCTGGTCCTGCA
CAAAGAGGCCAACTGTTCCTCCCACTGGAACCACAGGCGCATCGCGCCCTCGAAGAATGT
GGCCACCAGGCAGAGC----------CGGGC-------------CGTCGGGTGACCTCCT
TCGCCAAGTCCACCGCCTGTTCTAGAAAGTAAGTCCCCGCCTGCCGCAGGAGCTTGTCTT
GAGATTCCGGATCCAAGCCGCAAGTCTACTTCAAGCTATGAGTACGACAGTCGTGGCACT
GATAACAAACGGCCCACGCCAGGCTGAGTAATCGCTACGGTCTGGCTGTAGTCTGTGGCA
----------------------------------GGAGTCGTCCTGTTCCGGGCACAGCT
GTCGTTTGGGGCATTTCATTTCGTAATGCGTTGCTGCCTTATTGGCATACATATTTTATC
ATTATCAAAGAAAAAATGGCTTACTAGCATTGTGTTTAGGCGAGCATTTCTTAATATTTA
ATTT--------------------------------------------------------
------------------------------------------------------------
```

TRGF 1 conserved region alignment (including 4 outgroup species, excluding D. melanogaster)

```
>DROSI-NT_479536.1-896197:897745
CGG--------------------------------------------GGAGGCGTCCCCC
GAAAGTAGGCAATCCACGGATGGGGGTGGATGGGTGCT------------TGGGTGCCT-
GGTGACCCCAGCCCACAGTGGGAGTACAATGTGGGCGGCATGACTCGGTAA-------TT
CCAGGCCCATCTGCGGCATTTTATGCTCCAATAAAATCGCAATGT---GGCCGCGAGCTT
AAAGCTTGATTG---GATCAGGAAAGGGACGTGGAACAGTGACCAGTCCACCATTGTTTC
ATTAGGTGCTCCCAGAAGAGTCAAGTACAGAGAATTTGCCA--AAGAATGACGACAATCT
GTGCTTGGGAGATTGAAACACTGTTCATTATAAGATCCCTTCTAGCTGTTCGCCCCATAA
ACCATCCGAAAATCTAGGGAGTTACAGAGCCTTCTTTAACGCATAAACTGAAAAGGCATA
TATCAACTGATGAACAGGACTAATAATCTTAATTAGGAATGAAGTGGACACCACTGACGA
TAACTGCAAAAAGTTGTACTTCAGCGATCTGCTGTGCAGCTCTATCGCGATGGATGGGCG
AATTGGTGGACACATTACTCATGTTCAGAACAAAGACATTATAATGTATAGAATATTCTG
TCTTCGGTCAGAATAAATGGTGTATTAGCCCACTACGACATAGAATATAAGGAAGATACC
AAAGCAGGTCAGGACTCTAGTGCTGGAACACGGTCAGAGGAAATACAGCTCCTGCTCCGT
-GATGTGGCAGGCCACATCCTCCT------CGTTTTCC------------AGCAAGCCGC
CAAGGAGCGAGAAATTCCGGAGCAGCTGCTCCAACTACAGTGTCATCTT-GAATGTGGGA
ATCTGAGTAATA---------------CACTAAATTAA----------------TAAGAC
AGGCTGGGCTAA-CGACTCCAAATTTACCTGAAGCACAACTATGCCGGCCGG--------
---CAAGATGACCGCAAAATTTGTGAACAGTCGGACCAAGACCAGGCCACAC-------C
CCAACAGCTGAA---------------------------CCACCGCAAGGGCGGCGGAGC
TC----------------------------------------------TGGAGTTGGTCC
TGCAGAAAGG-----CCAGCTGTTCCACCCACTGGAGCAACATGCCCAGCGCGTCCTCGC
A--------------------------CCACCATGGGCAG--------------ACCGGA
TGACCTTTT-GCCCAGATCCACCGCCCGTTCTGAAAAGGGGGTCCCCTACTGCGTCGGGA
GCATGTTCCGAGATT---------CCTGCTCCAAGCCGCAAGA-----------------
-------CTACTGCGAGCTAAGAGGATGACAGTCGTGGCAGTGATAACAAA---------
--------CGGCGTACGCCAGGCTGAGTAATCGGTCCGGTCTGCCTTTAGTCTGTGGCAC
ATCCTGGAGAG------------------------------ATTGCTTCGCTACGAGGCG
TCGTAAGAGGTCCTTTTCCGGGCACAGCTGTCGTTTCGGAAATTTCATTTCGTAATGCGC
TCCTGTCCTATTAACATACAT--ATTGTACCATCTTCGTAGAAAAACTGATTTAG-----
---------ATGAATTAT------------------------------------------
------------------------------------------------------------
------------ATTTAAAAGAATTTT---------------------------------
------------------------------------------------------------
------------------------------------------------------------
```

-------------------------------TTTAAATATGAAATTTGTAG---------

------CCTAAGCTAACTTGAAA-------------------------------------

---TTGGTCCGGAATCCCA---
-----------------------GTCATCCGATGGCATCACTGCTTT-------------

-----TGAAGAGCGTTTCTCTACACAGATAACCATCAAAATATATCCCAAAATGAACCTA

>DROSE-NW_001999695.1-1113339:1114909
CGG--GGAGGCGTCCCCC
GAAAGTAGGCAATCCACGGATGGGGGTGGATGGGTGCT------------TGGGTGCCTG
GGTGACCCCAGCCCACAGTGGGAGTACAATGTGGGCGGCATGACTCGGTAA-------TT
CCAGG-CCATCTGCGGCATTTTATGCTCCAATAAAATCGCAATGT---GGCCGCGAGCTT
AAAGCTTGATTG---GATCAGGAAAGGGACGTGGAACAGTGACCAGTCCACCATTGTTTC
ATTAGGTGCTCCCAAAAGAGTCAAGTACAGAGAATTTGCCA--AAGAATGACGACAATCT
GTGCTTGGGAGATTGAAACACTGTTCATTATAAGATCCCTTCTAGCTGTTCGCCCCATAA
ACCATCAGAAAATCTAGGGAGTTACAGA-CCTTCTTTAACGCATAAACTGAAAAGACATA
TATCAATTGATGAAAAGGACTAATAATCTTAATTAGGAATGAAGTGGACACCACTGACGA
TAACTGGAAAAAGTTGTACTTCAGCGATCTTCTGTGCAGCTCTATCGCGATGGATGGGCG
AATTGGTGGACACATTACACATGGTCAGAACAAAGACATTATAATGTATAGAATATTCTG
TCTTCAGTTAGAATAAACGGTGTATTAGCCCACTACGACATAGAACATAAGGACGATACC
AAAGCAGGTCAGGACTCTAGTGCTGGAACACGGTCAGAGGAAATACAGCTCCTGCTCCGT
-GATGTGGCA---CACATCCTCCT------CGTTTTCC------------AGCAAGCCGC
CAAGGAGCGAGAAATTCCGGAGCAGCTGCTCCAACTACAGTGTCACTTT-GAATGTGGGA
ATCTGAGTAAAT---------------CACTAAATTTAA---------------TAAGAC
AGGCTGGGCTAA-CGACTCCAAATTTACCTGAAGCACAACTATGCCGGCCGG--------
---CAAGATGACCGCAAAATTAGTGAACAGTCGGACCAAGACCAGGCCACAA-------C
CCAACAGCTGAACCACCAGATGATTGAGCAGGACTACAGCCACCGCAAGGGCGGCGGAGC
TC--TGGAGTTGGTCC
TGCAGAAAGG-----CCAGCTGTTCCACCCACTGGAGCCACATGCGCTGCGCGTCCTCGC
A--------------------------CCACCATGGGCAG--------------ACCGGA
TGACCTTTT-GCCCAGATCCAACGCCCGTTCTGTAAAGGGGGTCCCCTACTGCGTCAGGA
GCATGTTCCGAGATT---------CCTGCTCCAAGCCGCAAGA-----------------
-------CTACTGCGAGCTAAGAGGATGACAGTCGTGGCAGTGATAACAAA---------
--------CGGCGTACGCCAGGCTGAGTAATCGGTCCGGTCTG-CTTTAGTCTGTGGCAC
ATCGTGGAGAG------------------------------ATTGCTTCGCTACGAGGCG
TCGTAATAGGTCCTTTTCCGGGCACAGCTGTCGTTTCGGAAATTTCATTTCGTAATGCGC
TCCTGTCCTATTAACATACAT--ATTGTACCATCTTCGTAGAAAAACTGATTTAG-----
---------ATGAATTAT--

------------ATGTAAAAGAATTTT---------------------------------

-------------------------------TTTAAATATGAAGTTTGTAG---------

------CCTAAGCTAAATTGCAA-------------------------------------

---TTGGTCCGGAATCCCA---
-----------------------GTCATCCGATGGCATCACTGCTTT-------------

```
-----TGAAGAGCGTTTCTCTAAACAGATAACCATCAAAATATATCCCAAAATGAACTTA
------------------------------------------------------------
-------------
>DROYA-NT_167065.1-1351799:1352543
------------------------------------------------------------
------------------------------------------------------------
------------------------------------------------------------
------------------------------------------------------------
------------------------------------------------------------
------------------------------------------------------------
------------------------------------------------------------
------------------------------------------------------------
------------------------------------------------------------
------------------------------------------------------------
------------------------------------------------------------
------------------------------------------------------------
-----------------------------------------------GCTCCTGCTCCGA
AGATGCGGCAGGCCTAAGCCTCCT------TGTCCTCCACATGCAGGAGCAGAAGGAGC-
CAAGGAGCGA---------------TAGTTTTAGCTGCAGTGTCACTTC-GTATCTGTTA
GCCTGTGTAAT-----------------ACTAAATTTAA---------------TGGGGC
AGGCTGAGCTCA-CGACTCCAAACTTAACTGAAGCAGAACTA------------------
--------------CGAGTATACCGGCCTGTAGGAGTAGGACCAGACC------------
------GCTGACCCAACA---TATTGAGCAGGACTACTGCC--------GCCGGTGGAGC
AC----------------------------------------------TGGATCTGGTCC
TGCAGAAAGA-----CCAGCTGTTCCACCCACTGG-ACCACATGCGCAGCGCGCCTTCGA
AGAAGGTGGCCACCAGGCAGAGATGAAACACCATGAGCGGG--GAGACGGCCCCGCCGGT
TGACTTGCC---CCATATCCACCGCCCTTTCTAG-AATGGGGTCCCAGCCTGCGTCAGGA
GCATGCTCTCAGATT---------CCGACTCCAAGCCGCAAGT-----------------
-------TCACTTTAAGCTATGAGTATGACAGTCGTGGCAGTGATAACAAA---------
--------CGGCCCACGCCAGGCTGAGTAATCGGTACGGTCTGCCTCTAGTTTGTGGCAC
ATCCTGAAAAG------------------------------ATTGCTGCGTTACGAGCCG
TCCTAAGTAGTCCTTTTCCGGGCACAGCTGTCGTTTCGGGAATTTCACTTCGTAATGCGT
TCCTGCCCTATTAACACACAT--ATTTTATCAT------AGAAAAAATGGGTTAT-----
---------TAGTATTGT------------------------------------------
------------------------------------------------------------
------------ATTTAAACGAACATT---------------------------------
------------------------------------------------------------
------------------------------------------------------------
-------------------------------TTTTAATATGAAATTT-------------
------------------------------------------------------------
------------------------------------------------------------
------------------------------------------------------------
------------------------------------------------------------
------------------------------------------------------------
------------------------------------------------------------
------------------------------------------------------------
------------------------------------------------------------
-------------
>DROER-NW_001956552.1-1266146:1266887
------------------------------------------------------------
------------------------------------------------------------
------------------------------------------------------------
```


---GCTCCTGTCCCGT
GGATGCGGCAGGCCACAGCCTCCTTGTCCCTGTCCTCCACATGCAGGAGCAGCAACCCG-
CAAGGAGCGAGAAATTCC-AAGCAGCAGCTTCAGCTGCAGTGTTACTTC-GCATCTGTGA
GCCTGAGTAAT-----------------ACTAACTTTGT---------------TAA---
-----------------TCCAAATTTACCTGGAGAAGCACTATGCGGGCCAG--------
---CAAGGTGACCGCAAAATTGGTGAACAGTCGGACTGAGACCAGGCCACAG-------T
CCAGCAGCTGACCAACCA---AAATGAGCAGGACTATGGCCACCACAAGGCCGGTGAAGC
AC--TGGAGCTGGTCC
TGCACAAAGAGG---CCAACTGTTCCTCCCACTGGAACCACAGGCGCATCGCGCCCTCGA
AGAATGTGGCCACCAGGCAGAGCCGGG-------------------------CCGTCGGG
TGACCTCCTTCGCCAAGTCCACCGCCTGTTCTAGAAAGTAAGTCCCCGCCTGCCGCAGGA
GCTTGTCTTGAGATT---------CCGGATCCAAGCCGCAAGT-----------------
-------CTACTTCAAGCTATGAGTACGACAGTCGTGGCACTGATAACAAA---------
--------CGGCCCACGCCAGGCTGAGTAATCGCTACGGTCTGGCTGTAGTCTGTGGCA-

---GGAGTCGTCCTGTTCCGGGCACAGCTGTCGTTTGGGGCATTTCATTTCGTAATGCGT
TGCTGCCTTATTGGCATACAT--ATTTATCATTATCAAAGAAAAAATGGCTTAC-----
---------TAGCATTGT--

------------GTTTAGGCGAGCATT---------------------------------

-------------------------------TCTTAATATTTAATTT-------------

>DROAN-NW_001939291.1-23186469:23187071

--GAGACCGGCTCTTGCTGCAT
ACATTCTGTAGCAGGAGGCCTCCT------TGTTCTCCACATGCAGCAGCAGTAGTCCCC
CCAGCAGCGCGAAAACCCTCAAAATCAGTTGGAGTTGCAACGGCACTGC-ATAAACATGG
GCCTGGATCAG-----------------AGTTAATTGAAGTTGATGCGCCCTCTTGGGCG
ATGTGTGGCGGG-CAAATGTGTGCTTACCTGCAGCAGGACGATGCCGGCCAG--------
---AAGGGTAACCGCAAAATTAATAAACAGATGCAGCCACACAAGCACACAG-------C
CCACCAGCTGGCCCACCA---GAAGAAACAAGGCAACAGCCACGGCCAGGTCGCCGGAAC
AC--TGTATTTGGTCC
TGCAGGAAGT-----TAATCTGCACCATCCACTGAAAAAACATGCGCAAGGCGTCCTCGA
AGAAAGTGGCTACAAGGCAGAAGCGGGCCACCACAGGCAGAGTGGGTCGACACCGGCGGA
TGACCTCCTCGCCCAGGTCCTCCGCCTGGCGTAGCCATCGGGTC----ACCATGTCTGGA
GCTTGTGGCCAGG-----------------------AGCAAAG-----------------
-------TGATTTCTCTCTCCGGGCGTGTCAGTTGCGGCGGCAATAACAAA---------
--------CAGCGACTGCCACTCTGAG---------------------------------

>_R_DROSU-NW_016027187.1-205755:207990
AAGTGCCTCTGCAACTGAAGCTGCTGCTCCGGAATTTCTCGCTCCTTGGAGGCTTACTGC
TACTGCATGTGGAGAAC--AAGGAGGCGGTGGCATGCCGCATCTACGGAGCAGGAGCTGG
ACTGCCTTTTGTTGTGAGCCGGCGTTCCCAGCATCTGATGCAGCTCACTGGAAGAATCCT
GCTGGCCCTCATGTATCTTACCCTCTTCCAGCAGTACTTCACCATTCCAGCCATGGTCTT
GAACTCTTTTGGCCTGATCCTGATGGCCATATTAA------------GCAATGTTGATTG
TTTGGTAACTATACAAATTGTATTGTAC------TTTACCATTAAAAGTGGTTGT-ATAT
GTATTTG---------------------------------------TTTTCGCTTTGTAT
AGAACTTAACAGTTT--------------CTTGTTGCAATAGGTTCATCGTAATGGGCTA
TCGCACCCGAATGGCTGCTTT----------------------GTGTCTTTCGCTAATTC
TCACCGTGTGGA---ATGTGACCACAAACGCCTGG--TGGTTCGACG-------------

---ATGGAGATGCT
AGG----------------------------GATCTGCTGAAGTACAACTTCTTTCACAC
CTTATCGGTGGTAGGAGGTCTTCTGATGGTTGTGGTTCTTGGACCTGGGGAAGTATCCCT
CGAGCAATATAAAAAA---CAGTGGTAGCTTGAGTTGGTGTATTTTTACAGTAATCGAAA
AGAGGAGTGATTCAAGTTATCAAAAGCGATTAAAAAAATATTGA----------TGATAT
AGGCTTGGTGAATCTTTTTACATTGACTTGACTGAATGGTATACTTAACAGTTTCTTGT

TGCAATAGTAAATAGTTAATTAACTATCAAAAATAGTTATAGTTACATAAGATCTCTCTC
TCATAAGATCTTCCACAC---AAAAGTATGCAACAATAACTTTAAAAATGTTCTCCGTTC
ACCTAGACTCCCAAGCCGAACTCTGAAAAGTTAAGATTTTACTTTAAACATGTATTCTCT
TAAAAAAAGTAAACCTAAACGTATTTTTCCACTGG-----------CTGGATGTCTTTTA
TCAAAAGTGCAATTTGA---
-AAATTACTTGTTTTTATTTTGCTTTTAAACTAG--------------CATGCTACAGTG
ACGTATCTATAATTTAATTAGTAACTTTATCAATTTTGCAGGTATATATTTACCATAAAA
ACATCACCAACTATATTTTATGGGTTGGATAACTATATAAATAGTTATATAACTACAATC
TGAGGGTTGAATCCATTCTACACTGTTTAATAGTCACA---TAACTATATTTTATAGTAA
CTCTTACGTAAAATTGACAATTTGATGGTTGGGGCCCTTTTATTATTATATTGGTAAATT
TTACCAATCGATTTTTTTTGTGTGTA---------TAAACAAGTTCAATACCAAATTTAT
GACAGCTCAACTTTTATAAAGTAATTTTGATAAAACTTTAAAAAGAACGGCTTGCCGCCT
TCTAAAATTTAGCGTTGTTTCTTTTCATATGCTAAGTAATATAAAACAATTCAAATATAA
AAAAGATATTTTTCTTGCGGCTGTCTATATAAATTAACAATTTTAATTCACAAAAGGGGC
CGTCCATAAAGCACGTGGACAAATTTTAATCACTTAACCTAAACCTAACCTACTGGCGTC
ACATTGTCCACTTTTAGCACTACAATACTTTTGTTCGAAAAACCATTTCAGAGTGGTGTA
ACCCCTTGCAAAACCTTGACTTAGAAATATAAACAACACTTTTTGTTTAAAATTATTGAG
AAAATATGTATGTTAGCAAGGGCAGTGTGCATTATGATATGAAATTCACAGAACTGCAGC
TGAGCCCGGCAGAGGACCATGTTGGGCGCCTCTTAACAGAGCAATCTTTTCAAGAAGTGC
CACAGACTCGAGACAGACCGGAACGATTACTCAGAGTGGCACTGGCCGTTTGTTATCACT
GCCACGCCTGTCAGCCAGGGACGCCGAGAGAAATCACTTTGCTTGTAAGTGGAGTTTGCG
GCTTGGAGCAGGAATCTCAGGACATGCTGGTGACCCAGACGGGAGCCCGATGGCTGCGCA
GGGCGGAAGATTTGGGGGAGGAGGTCGCCCGGCGGTGCCGCCCCTTTCTGCCCTCGGTGG
CCCGCTTCTGCCTGGTGGCCACCTTCTTCGAGGACGCCCTGCGCATGTGGTTCCAGTGGG
TGGAACAGCTGGCTTTCCTCCAGGACCACCACCA---------GTGCTCAGACGGCCTTG
CGGTGGCAATAGTCCTGCTGAATCTGGTGGGTCAGCTGGTGGGCTCGGCTCTGGTCATCC
TGCACCTGTTCAC
>_R_DROPS-NC_009005.2-21825514:21826183

---C
AAAGCTGTTGAG---GACCACGGCGGCGAGGCCGAAGTACTGCTGTAGCACTGTCAGATA
CATGAGGGCCAGCAGGA---
--TCCGTCCCGTGA
GCTGCATCAGG---

-------------------TGCTGCGATCGCCTGGGCTCCTCCA----------------

---------------------------------GCAGCGGAAGGCCTGCGCGCGCTCCAT
AGATCCGACAGGAGGAGGCCGCCTCGTCATTGGCCTCCACCAGTAGCAGAAGCAGTCCGC
CAAGCAGGGAGAAGTTTCGCAGCATCTGCTCCAGTTGCAGGGGCACAGA-GTAGACGTG-

-------------------------CAACTGGAAGAGCACCAGGCCGACCAG--------
---CAGTGCCACGGCCATATTGGTCCACACTCGGAACAAGACCAGACCGCAG-------C
CCACCAGCTGACCCAACA---GATTGACCTGGCAGCTCAGGAAGGCCCCGCCCCAGGAGA
AA--TTCAAGTGGATC
TGGAGGAAGG-----AGATCTGGTCCTCCCACTGGCACCACATGCGCAGGGCGTCCTCGA
AGAAGGTGGCCACCAGACAGAGGCGCGCCACTGTGGGCAGCACGAGACGACTCCGCCGGA
CGACCTCATCGCCCATATCCTCCGCCCGGGCCAGCCAGTCTGTC----CACATTTTGGTC
ACTTCCATGTCGGAG---------CTGCT-------CGGAGAA-----------------

-------GGAAGTCCGTTTCTGGTTGCGGAAGTGGTGATATATACAAAAT----------
--------GAGC--

---AATGAGT

>DROMI-NC_030304.1-9388218:9388887

AAAGCTATTGAG---CACCACGGCGGCGAGGCCAAAGTACTGCTGAAGCACTGTCAGATA
CATGAGGGCCAAAAGAA---
--TCCTTCCCGTGA
GCTGCATCAGG---

-------------------TGCTGCGATCGCCTGGGCTGCTCCA----------------

---------------------------------GCAGCGGAAGGCCTGCGCGCGCTCCAT
AGATCCGACAGGAGGAGGCCGCCTCTTCATTGGCCTCCACCAGTAGCAGAAGCAGTCCGC
CAAGCAGGGAGAAGTTTCGCAACAGCTGCTCCAGTTGCAGAGGCACAGA-GTAGACGTG-

-------------------------CAACTGGAAGAGCACCAGGCCGACCAG--------
---CAGTGCCACGGCCACATTGGTCCACACTCGGAACAAGACCAGACCGCAG-------C
CCACCAGCTGACCCAACA---GATTGACCTGGCAACTCAGGAAGGCCCCGCCCCAGGAGA
AA--TTCATGTGGATC
TGGAGGAAGG-----AGATCTGGTCCTCCCACTGGCACCACATGCGCAGGGCGTCCTCGA
AGAAGGTGGCCACCAGACAGAGGCGCGCCACTGTGGGCAGCACGAGACGACTCCGCCGGA
CGACCTCATCGCCCATATCCTCCGCCCGGGACAGCCAGTCTGTC----CACATTTTGGTC
ACTTCCATGTCGGAG---------CTGCT-------CGGAGAA-----------------
-------GGAAGTCCGTTTGTGGTTGCGGAAGTGGTGATAAATACAAAAT----------
--------GAAC--

---AATGAGT
T---

TRGF 2 sequences

>Dsim_GD20667
ATGCCTCCAGCTGGGCTGGCCCCTCGTCCTTCGCTTCATATCCTGTCAGGAGCTGACTTTTT
CCTGTTGCCGCTGCTTCCTTCCTTCCTTTCTTCTGGCTCTTATCTTCGTTTAACTTTCTACC
ATGTTATGGCCCGTTATCTGTCTGCCCGTGTACGTGTCCTTCGTGTCGGTGTGTGTTCGGTT
TCTATGTGTGTGTGTGTGTATGTGTGTGCTTCCTCGTCTCTGTTTGCATTTTTGAGCAAATT
AAGTGAAATTACATTTTTCTATGCGATGCTCTTAATGTTATTTGCATACGTATCCTGCAGGC
TACAATATTTACAAATGAATGCAGTCCGGTGCCGTTCGCTTGGTCCTTGGTCCTCGGTCCTT
GGGGGCTTGGTCCTTTCGAGAATCAGACAAGCGGCTGTTGAACCAACCACCTTAACTGCCCA
ACTTCCCAGCAGACCAACCGTCCAGCCGAACTAA
>Dsec_GM19408
ATGCCTCCAGCTGGGCTGGCTCCTCGTCCTTCGCTTCATATCCTGTCAGGAGCTGACTTTTT
CCTGTTACCGCTGCTTCCTTCCTTCCTTTCTTCTGGCTCTTATCTTCGTTTAACTTTCTACC
ATGTTATGGCCCGTTATCTGTCTGCCCGTGTACGTGTCCTTCGTGTCGGTGTGTGTTCGGTT
TCTATGTGTGTGTGTGTGTATGTGTGTGCTTCCTCGTCTCTGTTTGCATTTTTGAGCAAATC
AAGTGAAATTACATTTTTCTATGCGATGCTCTTAATGTTATTTGCATACGTATCCTGCAGGC
TACAATATTTACAAATGAATGCAGTCCGGTGCCGTTCGCTTGGTCCTTGGTCCTTGGGGGCT
TGGTCCTTTCGAGAATCAGACAAGCGGCTGTTGAACCAACCACCTTAA

TRGF 2 conserved region alignment

>Dsim-NT_479536.1-13995750:13997250
ATGACCTTATGTATCACCCTAACGATTTCCATTTGCTCCCTT------------------
CCCGCTCCAGTCGTCGTATCGAAGTAACTGCCTACTTCCTCGATGTCCTCGAATGCC-
TCCAGCT----
GGGCTGGCCCCTCGTCCTTCGCTTCATATCCTGTCAGGAGCTGACTTTTTCCTGTTGCCGCT
GCTTCCTTCCTTCCTTTCTTCTGGCTCTTATCTTCGTTTAACTTTCTACCATGTTATGGCCC
GTTATCTGTCTGCCCGTGTACGTGTCCTTCGTGTCGGTGTGTGTTCGGTTTCTATGTGTGTG
TGTGTGTATGTGTG-----
TGCTTCCTCGTCTCTGTTTGCATTTTTGAGCAAATTAAGTGAAATTACATTTTTCTATGCGA
TGCTCTTAATGTTATTTGCATACGTATCCTGCAGGCTACAATATTTACAAATGAATGCAGTC
CGGTGCCGTTCGCTTGGTCCTTGGTCCTCGGTCCTTGGGGGCTTGGTCCTTTCGAGAATCAG
ACAAGCGGCTGTTGAACCAACCACCTTAACTGCCCAACTTCCCAGCAGACCAACCGTCCAGC
CGAACTAA
>Dsec-NW_001999689.1-14856944:14858464

ATGACCTTATGTATCACCCTAACAATTTCCATTTGCTCCCTT------------------
CCCGCTCCAGTCGTCGTATCGAAGTAACTGCCTACTTCCTCGATGTCCTCGAATGCC-
TCCAGCT----
GGGCTGGCTCCTCGTCCTTCGCTTCATATCCTGTCAGGAGCTGACTTTTTCCTGTTACCGCT
GCTTCCTTCCTTCCTTTCTTCTGGCTCTTATCTTCGTTTAACTTTCTACCATGTTATGGCCC
GTTATCTGTCTGCCCGTGTACGTGTCCTTCGTGTCGGTGTGTGTTCGGTTTCTATGTGTGTG
TGTGTGTATGTGTG-----
TGCTTCCTCGTCTCTGTTTGCATTTTTGAGCAAATCAAGTGAAATTACATTTTTCTATGCGA
TGCTCTTAATGTTATTTGCATACGTATCCTGCAGGCTACAATATTTACAAATGAATGCAGTC
CGGTGCCGTTCGCTTGGTCCTT-------
GGTCCTTGGGGGCTTGGTCCTTTCGAGAATCAGACAAGCGGCTGTTGAACCAACCACCTTAA
CTGCCCAACTTCCCAGCAGACCAACCGTCCAGCTGAACTAA
>R_Dmel-NT_033777.3-11270425:11271990
ATGACCTTATGTATCACCCTAGCGATTTCGATTTGCACCCTT------------------
CTCGCTCCAGTCGTCGTATCGAAGTAACTGCCTACTTCCTCGATGTCCTCGAATGCC-
TCCAGCT----
GGGCTGGCCCCTCGTCCTTCGCTTCATATCCTGTCAGGAGCTGACTTTTTCCTGTTGCCGCT
GCTTCCTTCCTTCCTTTCTTCTGGCTCTTATCTTCGTTTAACTTTCTACCATGTTATGGCCC
GTTATCTGTCTGCCCGTGTACGTGTCCTTCGTGTCGGTGTGTGTTCGGTTTCTATGTGTGTG
TGTGTGTGTGTGTG-----
TGCCTCCTCGTCTCTGTTTGCATTTTTGAGCAAATCAAGTGAAATTACATTTTTCTATGCGA
TGCTCTTAATGTTATTTGCATACGTATCCTGCAGGCTACAATATTTACAAATGAATGCAGTC
CGGTGCCGTTCGCTTGGTCCTTGGTCCTCGGTCCTTGGGGGCTTGGTCCTTTGGAGAATCAG
ACAAGCGGCTGTTGAACCAACCACCTTAACTGCCCAACTTCCCAGCAGACCAACCGCCCAGC
CGAACTAA
>R_Dyak-NT_167065.1-11130997:11132591
ATGACCGTATGTATCACCCTACCGATTTTCATTTGCTCTCTTCCCCTCGAGTGGTTTACTGC
CCGCTCCAGTCGTCATATCGAAGTAACTGCCTACTTCCTCGATGTCCTCGAATGCCTTCCAG
CT----
GGGGTGGCCCCTCGTCCTTCGCTTCATATCCTGTCAGGAGCTGACTTTTTCCTGTTGCCGCT
GCTTCCTTCCTTCCTTTCTTCTCGCTCTTATCTTCGTTTAACTTTCTACCATGTTATGGCCC
GTTATCTGTCTGCCCGTGTACGTGTCCTTCGTGTCGGTGTGTGTTTGGTTCCTATGTGTGTG
GGTGTGTGTGTGTGTGTGCTGCCTCGTCTCTTCTGTTTGCATTTTTGAGCAAATCAAGTGAA
ATTACATTTTTCTATGCGATGCTCTTAATGTTATTTGCATACGTATCCTGCAGGCTACAATA
TTTACAAATGAATGCAGTCCGGTGCCGGGCGCTTGGTCCTTGATTCTCGGTCCTTGGGGGCT
TGGTCCTTTGGAGAATCAGACAAGCGGCTGTTGAACCAACCACCTTAACTGCCCAACTTCGC
AACAACCCAACCGCCCAGCCGAACTAA
>Dere-NW_001956552.1-14521861:14523460
ATGACCGTATGTATCACCCTACCGATTTCCATTTGATCCCTTCCCCTCGAGTGGTTTACTAC
CCGCTCCAGTCGTCGTATCGAAGTAACTGCCTACTTCCTCGATGTCCTCGAATGCCTTCCA-
CTTGGGGGGGGGGTCCTCTTCCTTCGCATCATATCCTGTCAGGAGTTGACTTTTTCCTGTT
GCCGCTGCTTCCTTCCTTCCTTTCTTCTCGCTCTTATCTTCGTTTAACTTTCTACCATGTTA
TGGCCCGTTATCTGTCTGCCCGTGTACGTGTCCTTCGTGTCGGTGTGTGTTCGGTTCCT---
----
GTGTGTGTGTGTGCTTCCTCGTCTCTTCTGTTTGCATTTTTGAGCAAATCAAGTGAAATTAC
ATTTTTCTATGCGATGCTCTTAATGTTATTTGCATACGTATCCTGCAGGCTACAATATTTAC
AAATGAATGCAGTCCGGTGCTGTGCGCTTGGTGCTTGATTCTCGGTCCTTTGGGGCTTGGTC
CTTTGGAGAATCAGACAAGCGGCTGTTGAACCAACCACCTTAACTGCCCAACTTGCCAACTA
CCCAACCGCCCAGCC-AACTAA
